# Heroin-Induced Non-cardiogenic Pulmonary Edema: A Rare but Serious Complication

**DOI:** 10.7759/cureus.81548

**Published:** 2025-03-31

**Authors:** Yasser Hegazy, Piyush Puri, Ramsha Durrani, Santino E Patrizi, Muhammad Ghallab

**Affiliations:** 1 Internal Medicine, Icahn School of Medicine at Mount Sinai, Queens Hospital Center, New York, USA; 2 Medicine, Liaquat University Hospital, Hyderabad, PAK; 3 Internal Medicine Program, Icahn School of Medicine at Mount Sinai, Queens Hospital Center, New York, USA; 4 Internal Medicine, Icahn School of Medicine at Mount Sinai, NYC Health and Hospitals, Queens, New York, USA

**Keywords:** heroin, heroin abuse, non-cardiogenic pulmonary edema, opoid use disorder, severe pulmonary edema

## Abstract

Non-cardiogenic pulmonary edema (NCPE) is a rare but serious complication of heroin overdose, more frequently observed in fatal cases. This report discusses a 33-year-old male patient with a history of hypertension and heroin use disorder who presented with dyspnea and hypoxia. Upon examination, his vital signs indicated an SpO_2_ of 82% on room air, along with tachycardia and tachypnea. The patient disclosed heroin use the day before admission, and urine toxicology screening confirmed the presence of opioids and methadone. Pulmonary auscultation revealed bilateral crackles, while chest imaging, including chest X-ray (CXR) and computed tomography angiography (CTA) of the chest, revealed diffuse bilateral airspace opacities consistent with pulmonary edema, effectively ruling out pulmonary embolism. Arterial blood gas analysis indicated acute respiratory acidosis, which improved with oxygen therapy. A prior echocardiogram had shown normal cardiac function. Notably, diuretics were not administered, and the patient's oxygen requirements decreased within two days. NCPE is a diagnosis of exclusion, often characterized by persistent hypoxia following the resolution of opioid-induced respiratory depression, with radiographic evidence of pulmonary edema. The condition typically resolves within 24-48 hours with supportive care, as observed in this case. While NCPE is frequently identified during autopsy in heroin-related fatalities, its underlying pathophysiology remains poorly understood. NCPE should be considered in the differential diagnosis of respiratory failure in patients with heroin overdose. Given its potentially fatal consequences and the limited understanding of its pathophysiology, further research is warranted to optimize management strategies.

## Introduction

Heroin abuse represents a significant global public health challenge, contributing to substantial social, legal, and economic burdens. It has been associated with complications affecting the respiratory, nervous, and circulatory systems [1-3]. Its increasing prevalence poses challenges not only to healthcare systems but also to social, judicial, and economic sectors. The drug can be administered through intravenous injection, insufflation, or smoking. Heroin overdose is typically diagnosed clinically based on altered mental status, significantly reduced respiratory drive, pinpoint pupils, and supporting evidence of drug use [4]. Research indicates that its respiratory effects extend beyond respiratory depression to include asthma exacerbation, varying degrees of respiratory distress up to non-cardiogenic pulmonary edema (NCPE) [3]. NCPE is a less commonly acknowledged yet potentially life-threatening complication. First reported by William Osler in 1880, heroin-induced NCPE (HI-NCPE) has a poorly understood pathophysiology, with mechanisms hypothesized to involve alveolar-capillary membrane disruption, immune-mediated reactions, or hypersensitivity to heroin and its contaminants [1]. NCPE typically manifests as persistent hypoxia following the resolution of opioid-induced respiratory depression, accompanied by frothy, pink-tinged pulmonary secretions and a characteristic radiographic pattern of diffuse, fluffy pulmonary infiltrates. In some cases, patients may require mechanical ventilation to manage severe hypoxia. However, symptoms generally resolve quickly with supportive care, typically within a few hours to one or two days [5]. We present a case of a 32-year-old man who developed severe hypoxia and diffuse pulmonary infiltrates following heroin use. His condition significantly improved and fully resolved within 48 hours with supportive care alone. This case underscores the complexities of diagnosing and managing HI-NCPE while also exploring its underlying mechanisms and implications for clinical practice.

## Case presentation

Our patient was a 32-year-old man with a past medical history of IV heroin abuse, anxiety, and hyperlipidemia. He presented to the emergency department after waking up on the floor of his bathroom several hours after using heroin. Upon awakening, he experienced chest pain and a sensation of suffocation. Additionally, he had multiple episodes of hemoptysis and coughing, totaling less than one cup of blood. The patient reported that he had taken a larger dose of heroin that day, which is likely the reason for his loss of consciousness. Upon arrival, he required a non-rebreather mask at 15 L to maintain a saturation over 90%, as his SpO_2_ was 66% on room air. On examination, the patient was alert and oriented but in mild respiratory distress with bilateral rales. Cardiac examination showed sinus tachycardia with normal s1 and s2 and no murmur or additional sounds. The remainder of the examination was unremarkable. Arterial blood gas analysis revealed severe acidosis, with a CO₂ level of 71 mmHg and a lactic acid level of 4.1 mmol/L. An EKG showed sinus tachycardia with nonspecific T-wave abnormalities, and Troponin T was negative. A urine drug screen was positive for opiates. Complete blood count showed no leukocytosis, mild normocytic anemia, and platelets within normal ranges. The procalcitonin level was elevated, while blood and sputum cultures were negative. Point-of-care testing for COVID-19 and influenza was negative. Liver function tests showed mildly elevated transaminases (Table 1).

**Table 1:** Laboratory findings on admission. POC: point-of-care.

Laboratory test	Result	Reference range
PH	7.20	7.32–7.73
PCO_2_	71 mmHg	41–54 mmHg
HCO_3_	24 mmol/L	22–29 mmol/L
PO_2_ (on non-rebreather O_2_ mask at 15 L)	93 mmHg	83–108 mmHg
Anion gap	15 mEq/L	8–16 mEq/L
Lactic acid	4.5 mmol/L	0.6–1.4 mmol/L
White blood cells	9.6 × 10⁹/L	4.8–10.80 × 10⁹/L
Hemoglobin	13.2 g/dL	14.0–18.0 g/dL
Platelet	209 × 10⁹/L	150–450 × 10⁹/L
ALT	79 U/L	0–41 U/L
AST	56 U/L	5–40 U/L
Troponin T	<0.010 ng/mL	≤0.010 ng/mL
Procalcitonin	15.84 ng/mL	0.02–0.10 ng/mL
Drug screen test	Positive opiate	Negative
Blood culture	Negative	Negative
Sputum culture	Negative	Negative
POC COVID test	Negative	Negative
POC Influenza test	Negative	Negative

**Figure 1:**
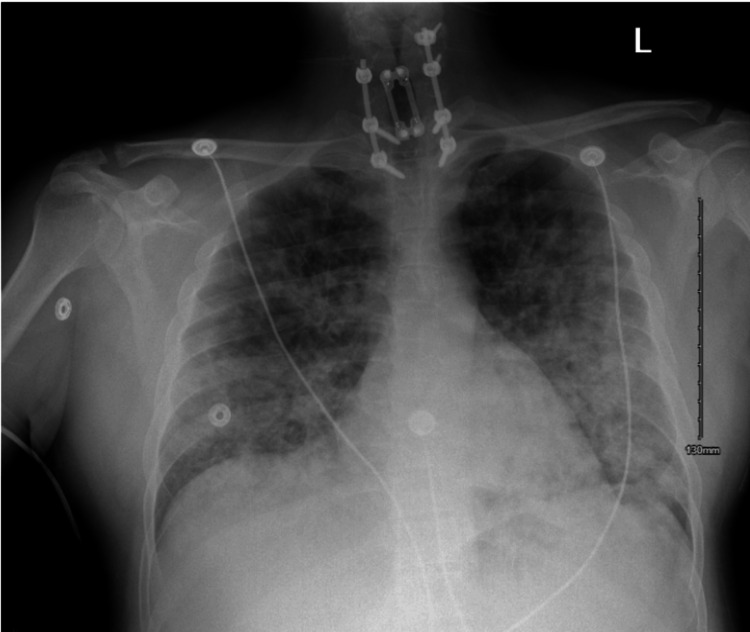
Anteroposterior chest X-ray showing diffuse, bilateral patchy alveolar infiltrates.

**Figure 2:**
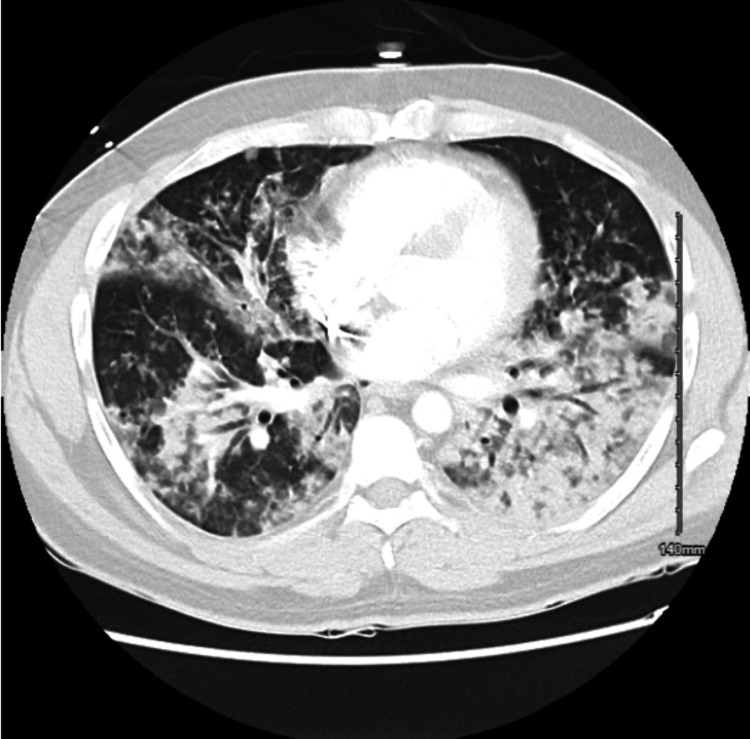
CT angiography of the chest in transverse view demonstrating multiple bilateral nodular opacities and consolidations with air bronchograms. No pleural effusion or pneumothorax present.

Chest X-ray (CXR) showed diffuse, bilateral patchy opacities (Figure 1). CT angiography of the chest was negative for pulmonary embolism but notable for innumerable bilateral pulmonary nodules and bibasilar consolidations (Figure 2). A previous echocardiogram revealed normal cardiac function. Based on imaging findings, physical exam, lack of leukocytosis or fevers, and normal echocardiogram findings, we believed it was more likely a drug-induced lung injury causing NCPE. He was started on oxygen supplementation, nebulization therapy, and antibiotic coverage for possible superimposed pneumonia with improvement in oxygenation. After two days of therapy and pulmonary hygiene, oxygen supplementation was de-escalated, and he was saturating normally on room air. The patient was near his baseline at this point and was discharged home.

## Discussion

Heroin is one of many drugs with increasing rates of abuse. It can be consumed through intravenous injection, snorting, or smoking. Diagnosing a heroin overdose involves recognizing signs such as reduced consciousness. Research has highlighted that heroin's effects on the respiratory system extend beyond respiratory depression, including asthma aggravation, pulmonary edema, and varying degrees of respiratory distress [3].

NCPE is a rare complication of heroin overdose. Its exact cause remains unclear, but it has been linked to both heroin use and naloxone administration. HI-NCPE was first described by William Osler in 1880. Despite extensive investigation, the pathophysiology of HI-NCPE is still not well understood and seems to be more complex than is usually suggested. Damage to the alveolo-capillary membrane and increased permeability of the vascular endothelium, mainly due to hypoxia, have been considered the final pathway of this edema, but several additional hypotheses have been proposed [3,6]. Studies suggest that the protein composition of edema fluid in affected individuals is similar to serum, indicating capillary leakage. Reduced levels of immunoglobulin M (IgM) and complement components in heroin users with pulmonary edema support an immune-mediated mechanism, potentially involving immunoglobulin deposition in the lungs [7]. Histamine release has been implicated as a mediator; it was thought that histamine release resulted in large gaps in the endothelium of bronchial venules leading to the edema. Arslan et al. reported that baseline histamine concentrations were high in chronic intravenous heroin users [3]. Mell et al. suggested that pulmonary oedema was due to naloxone-precipitated withdrawal and associated catecholamine surge. In opposition to this suggestion, Arslan et al. concluded that the lower rates of HI-NCPE observed in the most recent studies, rather than in the earlier ones, is the result of administrating naloxone at an earlier stage and more liberally [3].

Variations in heroin purity and the presence of additives further complicate its pathophysiology, with contaminants potentially contributing to hypersensitivity reactions or toxic effects [3]. Seasonal patterns in HI-NCPE cases, with higher incidences observed between April and July, suggest that factors beyond acute overdose may be involved, possibly hypersensitivity to heroin or its contaminants [8]. Autopsy findings in severe cases show systemic organ congestion, supporting the hypothesis of a shock-like hypersensitivity reaction rather than a direct overdose effect. Toxicological data also indicate that the severity of pulmonary edema may not correlate with the amount of heroin consumed, unlike other opiates [8]. Ultimately, while damage to the alveolo-capillary membrane and vascular endothelium appears to be the final common pathway, the development of HI-NCPE likely involves a complex interplay of hypoxia, immune responses, heroin composition, and other factors, highlighting the need for further research to clarify its mechanisms.

HI-NCPE is more common in males, individuals in their 30s, and those with relatively short histories of heroin use (average 2.9 ± 5.1 years). Patients are often found in an obtunded state (GCS < 8) by emergency medical services and typically require naloxone to support adequate respiration before hospital arrival. It typically manifests within two hours of drug intake and presents with symptoms such as rales, pink frothy sputum, severe hypoxia, and bilateral fluffy infiltrates on chest X-rays. Hemodynamic and pulmonary fluid analyses confirm its noncardiogenic nature. The absence of fever, rapid symptom resolution with supportive care, and quick improvement in chest X-ray findings in our case suggest that HI-NCPE is the most plausible diagnosis rather than underlying pneumonia [3,4]. Only one-third of affected individuals require intubation. The majority of patients need only additional oxygen, and their symptoms generally subside within 24 hours [3]. While most heroin overdoses historically occur in long-term users, less experienced users appear more susceptible to developing NCPE following an overdose. A suggested explanation is that although more experienced users are more likely to overdose, less experienced users can be prone to experiencing complications [6,9].

The optimal observation period for patients with acute heroin overdose should ensure that most cases of NCPE manifest during medical supervision. Assuming a higher-than-reported incidence of 10%, 95% of cases are estimated to occur within the first hour of observation. During a two-hour observation period, the likelihood of NCPE development is approximately 4.75%, with only 0.25% of cases presenting later. Extended observation (12-24 hours) appears unnecessary for most patients due to the low risk, although longer monitoring may be required for overdoses involving methadone or other oral opiates. Traditional guidance recommends observing patients with a history of respiratory arrest from opiate overdose to identify potential complications, including NCPE. This approach aims to refine understanding of the subset of heroin users at risk of developing NCPE following overdose [4,6].

## Conclusions

This case highlights the rapid onset and resolution of HI-NCPE, a rare but potentially fatal complication of heroin overdose. The patient presented with severe hypoxia and diffuse pulmonary infiltrates, which improved significantly within 48 hours with supportive care alone. Our case aligns with previous reports describing the rapid onset of HI-NCPE following heroin overdose, although the exceptionally swift resolution within 48 hours with conservative management alone appears less frequently documented in the literature. This underscores the variability in clinical course and highlights the need for further investigation into prognostic factors influencing recovery. In addition, it reinforces the importance of recognizing NCPE as a differential diagnosis in heroin overdose cases, particularly in patients with persistent hypoxia despite the resolution of opioid-induced respiratory depression. The underlying pathophysiology of HI-NCPE remains poorly understood. Given the increasing prevalence of heroin use and the potential for fatal outcomes, further research is needed to establish standardized diagnostic criteria, optimize management strategies particularly for severe cases requiring respiratory support, and evaluate potential long-term pulmonary outcomes.
